# Highly glycosylated MUC1 mediates high affinity L-selectin binding at the human endometrial surface

**DOI:** 10.1186/s12951-021-00793-9

**Published:** 2021-02-17

**Authors:** Lewis W. Francis, Seydou N. Yao, Lydia C. Powell, Sean Griffiths, Alexander Berquand, Thomas Piasecki, William Howe, Andrea S. Gazze, Mary C. Farach-Carson, Pamela Constantinou, Daniel Carson, Lavinia Margarit, Deya Gonzalez, R. Steven Conlan

**Affiliations:** 1 Swansea University Medical School, Singleton Park, Swansea, SA2 8PP Wales, UK; 2 Bruker UK Limited, Banner Lane, Coventry, CV4 9GH UK; 3 School of Dentistry, The University of Texas Health Science Center, Houston, 77054 Texas USA; 4 Department of Biosciences, Wiess School of Natural Science, Rice University, Houston, Texas 77251 USA; 5 Cwm Taf Morgannwg University Health Board, Princess of Wales Hospital, Bridgend, CF31 1RQ UK

**Keywords:** Adhesion, Single molecule force spectroscopy, Mucin, Implantation, Biophysics, Human reproduction

## Abstract

**Background:**

Sialyl-Lewis X/L-selectin high affinity binding interactions between transmembrane O-glycosylated mucins proteins and the embryo have been implicated in implantation processes within the human reproductive system. However, the adhesive properties of these mucins at the endometrial cell surface are difficult to resolve due to known discrepancies between *in vivo* models and the human reproductive system and a lack of sensitivity in current *in vitro* models. To overcome these limitations, an *in vitro* model of the human endometrial epithelial was interrogated with single molecule force spectroscopy (SMFS) to delineate the molecular configurations of mucin proteins that mediate the high affinity L-selectin binding required for human embryo implantation.

**Results:**

This study reveals that MUC1 contributes to both the intrinsic and extrinsic adhesive properties of the HEC-1 cellular surface. High expression of MUC1 on the cell surface led to a significantly increased intrinsic adhesion force (148 pN vs. 271 pN, p < 0.001), whereas this adhesion force was significantly reduced (271 pN vs. 118 pN, p < 0.001) following siRNA mediated MUC1 ablation. Whilst high expression of MUC1 displaying elevated glycosylation led to strong extrinsic (> 400 pN) L-selectin binding at the cell surface, low expression of MUC1 with reduced glycosylation resulted in significantly less (≤200 pN) binding events.

**Conclusions:**

An optimal level of MUC1 together with highly glycosylated decoration of the protein is critical for high affinity L-selectin binding. This study demonstrates that MUC1 contributes to cellular adhesive properties which may function to facilitate trophoblast binding to the endometrial cell surface through the L-selectin/sialyl-Lewis x adhesion system subsequent to implantation.
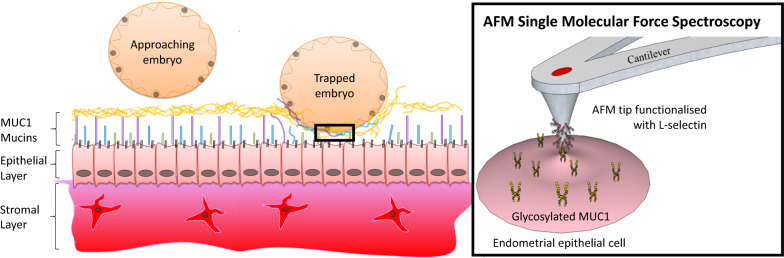

## Background

Mucins are high molecular weight O- and N-glycosylated proteins present on the surface of human epithelial cells found in the reproductive, bladder, respiratory and digestive tracts and can be secreted into the extracellular environment [1, 2]. Heavily glycosylated mucin structures act as barriers between the cell membrane and surrounding environment, shielding cells and tissues from toxins and proteolytic attack by bacteria and host proteases, and also function in cell adhesion, immune response and cell signalling [1–3]. Mucin protein glycosylation is initiated by glycotransferases, which catalyse the addition of N-acetylgalactosamine (GalNAc) through an O-glycosidic linkage to hydroxyl groups present on serines or theronines in the protein substrate [2]. The addition of GalNAc forms the foundation upon which highly ordered and complex oligosaccharide chains can be built through the addition of sugars including fucose, galactose, sialic acid and sialyl-Lewis X, leading to either linear or branched structures [2]. Sialyl-Lewis X is a mono-fucosylated oligosaccharide which functions as a ligand to a family of adhesion molecules, the selectins (P-, L- and E- selectins) [4]. Selectins are type-1 transmembrane glycoproteins which display binding affinity to glycans containing α2,3-linked sialic acid and α1,3-linked fucose residues [5]. Whilst P- and E- selectins are involved in immune recognition of the embedded embryo in the endometrium and trophoblast migration within decidual spiral arterioles, L-selectin and its binding ligands have been shown to play a crucial role in mediating the adhesion of the blastocyst to the endometrium [6].

The sialyl-Lewis X/L-selectin adhesion system has been implicated in many physiological processes, including leukocyte infiltration (of vascular endothelial cell surfaces), lymphocyte homing, tumour metastasis, and therefore is an important glycan mechanism in cell-cell interactions [4, 7, 8]. Sialyl-Lewis X/L-selectin interactions are required for successful implantation processes in the human reproductive system, mediating the adhesion of the embryo to the uterine endometrial epithelium surface [4]. Glycosylation patterns on proteins present on the human endometrium cell surface, including sialyl-Lewis X, are very dynamic, varying during embryogenesis, embryo implantation, invasion and placentation [4, 9]. Sialyl-Lewis X epitope levels at the endometrial surface peak during embryo implantation in healthy women [10], where blastocysts also demonstrate L-selectin expression [11, 12]. Also, in infertile women diagnosed with polycystic ovary syndrome (PCOS), endometriosis or Unexplained Infertility (UIF) L-selectin levels are altered, suggesting correct glycosylation is critical in implantation [10].

Previous studies have shown that the transmembrane mucins MUC1 and MUC16 are the two major mucins present on the surface of endometrial epithelial cells, whereas MUC4 is virtually absent, however their function in the reproductive process is yet to be fully resolved [13, 14]. The ability of the embryo to undergo the process of apposition at the endometrial surface appears to involve a delicate balance between adhesive and anti-adhesive properties of the surface glycocalyx; a mucin protein rich endometrial surface layer which may act as a natural barrier to the embryo attachment, except at the site of apposition [15–17]. *In vivo* studies in a transgenic mouse model showed that the loss of MUC1 at sites of implantation correlated with embryo attachment, however, in humans expression of MUC1 is high during peri-implantation indicating its possible role in this process [17, 18]. MUC1 can be decorated with sialyl-Lewis X in endometrial cells [4, 19] and expression of MUC1 in the infertile endometrium is significantly different than in fertile endometrium [20]. This suggests that MUC1 glycosylation in humans may promote initial recognition and adhesion events between MUC1 and the embryo, which could subsequently guide the embryo to the implantation site [20]. MUC16 also plays a role in modulating implantation, and loss of MUC16 has been shown to enhance trophoblast adherence *in vitro* [21]. Conversely, another study revealed an increase in endometrial MUC16 transcript levels in the receptive phase of fertile women, and a decreased transcript level in women with IVF failure [22]. Because of the known discrepancies between *in vivo* models and the human reproductive system and conflicting information within the literature, we developed an *in vitro* model to investigate the role of glycosylated MUC1 in L-selectin recognition in relation to the initial interaction of the blastocyst with the mucin layer extending far above the cell surface [13, 20].

In this study, we used single molecule force spectroscopy (SMFS) to probe endometrial epithelial cells to describe the nanoscale binding events that occur between L-selectin and mucin proteins. Using this *in vitro* assay we have been able to elucidate the adhesive and anti-adhesive roles of MUC1, and thus determine what appears to be an important role in the first critical stage in embryo recognition, crucial for human reproduction.

## Results

### Mucins facilitate intrinsic endometrial cell surface adhesion

**Fig. 1:**
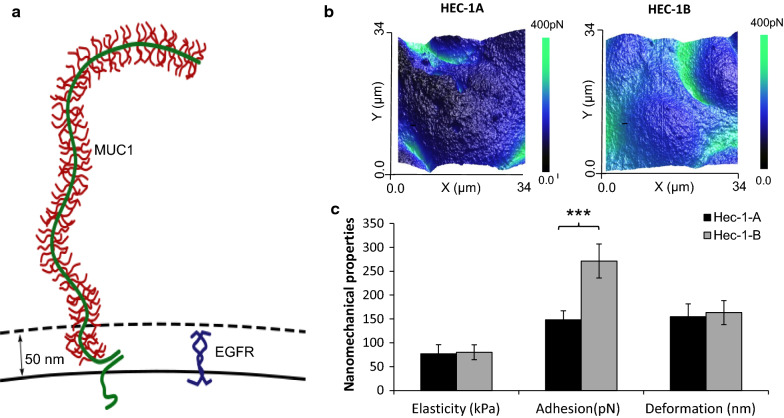
Endometrial epithelial cell mucins, mechanics and adhesion. **a** Schematic of transmembrane mucin MUC1 and Epidermal Growth Factor Receptor (EGFR) present on the endometrial cell surface, demonstrating MUC1 extension high above other surface receptor molecules. **b** AFM QNM surface adhesion images of HEC-1A and HEC-1B cell lines assessed with a non functionalized AFM probe. **c** Quantitative data of Cell Elasticity (kPa), Adhesion (pN) and Deformation (nm) from AFM QNM data from HEC-1A and HEC-1B cell lines with a non-functionalized probe. Data shown is based on a minimum of three biological repeats, with three cellular areas per repeat (n = 9), analysed as parametric data using 2 tailed T test. Significance given as *** p < 0.001

Mucins are important components of the endometrial cell glycocalyx layer and possess long elongated and flexible structures that extend well beyond the cell surface [23]. These mucins have a highly glycosylated central protein domain of tandemly repeating sequences, known as VNTRs (variable number tandem repeats), resembling an extended bottle brush like conformation (Fig. 1a). It is these highly glycosylated regions of the mucin strands which facilitate adhesive and anti-adhesive interactions on the cell surface [24]. However, the intrinsic and extrinsic adhesive role of MUC1 extending from the endometrial cell surface in successful implantation is currently disputed due to conflicting literature derived from *in vitro* and *in vivo* studies.

In this study, we performed AFM PFQNM mapping to quantify the intrinsic adhesive properties of HEC-1A and HEC-1B cell lines (Fig. 1b and c). The endometrial adenocarcinoma HEC-1 cell line was selected for this study as the cell line expresses MUC1 (HEC-1B is a substrain of HEC-1A) and has been widely used in implantation research [25]. While no differences in cellular stiffness and deformation were identified between HEC-1A and HEC-1B cell lines, a significantly increased adhesion force was measured for HEC-1B cells (271pN) when compared to HEC-1A cells (148pN, p < 0.001; Fig. 1b and c). Cell stiffness and deformation are mechanical properties linked to a cells sub-membranous cytoskeletal structure, while adhesion force is linked to the presence of membrane proteins on the cell surface [26, 27]. In this study, forces derived from the retraction part of the force curve measurements was used to assess the adhesive properties of the mucin layer extending from the cell surface. This data demonstrates that HEC-1B cell surfaces have a greater intrinsic adherence compared to HEC-1A.

**Fig. 2:**
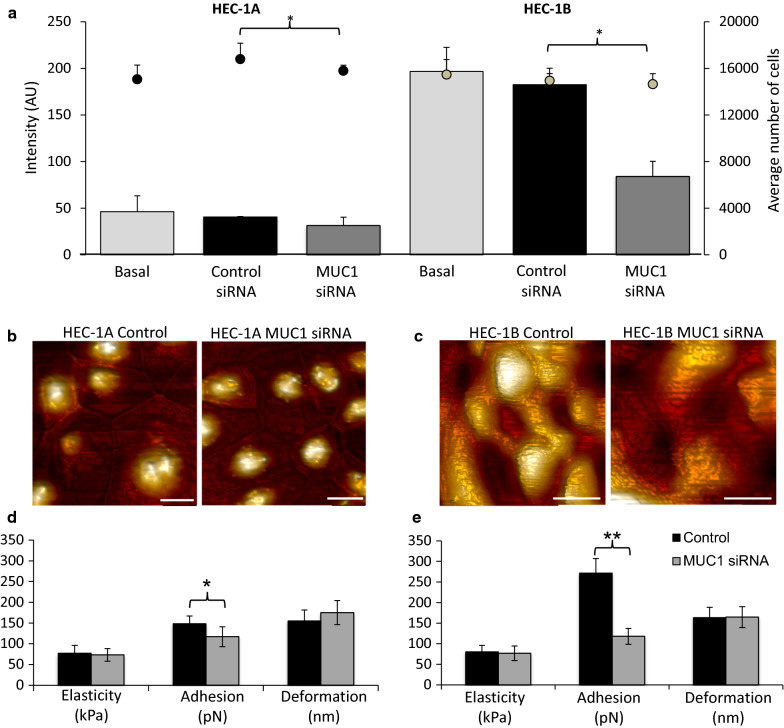
Cell adhesion of HEC-1A and HEC-1B cells in the presence/absence of MUC1 specific siRNA. **a** A population wide screen (minimum 1000 cells) assessed for the expression of MUC1 at the protein level using IN Cell microscope, in the presence and absence of MUC1 siRNA for both cell lines. **b** and **c** Topography AFM images of both cell lines in the presence and absence of MUC1 specific siRNA, scale bar 20 µm (**b** – control and MUC1 siRNA image Z range is 4.5 µm; **c** – Control image Z range is 8.8 µm, MUC1 siRNA image Z range is 4.0 µm). (**d**  and **e**) HEC-1A and HEC-1B cell mechanical and adhesive properties, stiffness (kPa), adhesion (pN) and deformation (nm), achieved with AFM QNM imaging were assessed in the presence and absence of MUC1 siRNA. All data shown is based on a minimum of three biological repeats with three cellular areas per repeat (n = 9), analysed as parametric data using 2 tailed T test. Significance given as * p < 0.05, **p < 0.001

To determine if the intrinsic adhesion properties of HEC-1 cells was linked to mucin expression we established a MUC1 specific siRNA knockdown model in HEC-1 cells. Protein expression analysis revealed that MUC1 protein expression was 3 times greater in HEC-1B cells (196 AU, Fig. 2a) compared to HEC-1A cells (46 AU). Also, MUC1 protein expression in HEC-1B and HEC-1A cells was significantly reduced by 53.9 % and 22.6 % respectively following MUC1 siRNA treatment compared to scrambled siRNA (p ≤ 0.05; Fig. 2a). AFM PFQNM imaging of the HEC-1A and HEC-1B cells revealed no topographical differences (Fig. 2b and c) and no significant differences in cell deformation and stiffness (p > 0.05; Fig. 2d and e) after MUC1 siRNA treatment. However, for the MUC1 abundant HEC-1B cell line the adhesion force was significantly reduced from 271 ± 35 pN to 118 ± 19 pN following siRNA mediated MUC1 ablation (p < 0.001). The adhesion force for HEC-1A cells, expressing less MUC1, was also significantly reduced, but to a lesser extent, from 148 ± 19 pN to 117 ± 24 pN following siRNA treatment (Fig. 2d; p < 0.05).

**Fig. 3:**
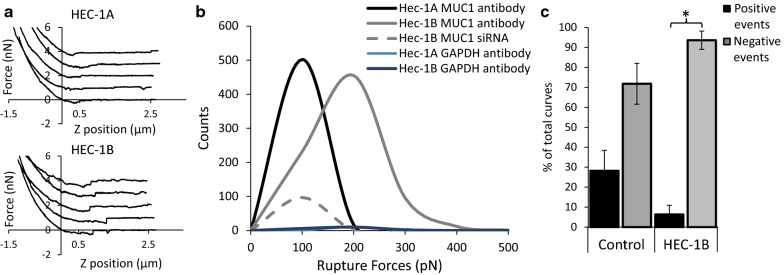
MUC1 VNTR specific antibody functionalised probe used to interrogate HEC-1A and HEC-1B cell surfaces. **a** Example SMFS force curves from HEC-1A and HEC-1B cell surfaces **b** the rupture force (pN) achieved from the surface of HEC-1A and HEC-1B cells achieved with a MUC1 antibody functionalized probe and an anti-GAPDH antibody functionalized probe, in the presence and absence of MUC1 siRNA. **c** The percentage number of positive and negative adhesion events assessed from the SMFS force curves achieved from HEC-1B cells in the presence and absence of MUC1 siRNA

To confirm that the reduction in adhesion force after MUC1 siRNA treatment was in fact due to MUC1 clearance from the surface of HEC-1B and HEC-1A cells, we used an AFM probe functionalised with an antibody specific for the MUC1 extracellular VNTR domain region (Fig. 3). The magnitude of rupture forces achieved between the AFM probe and cell surface were lower for HEC-1A compared to HEC-1B cells (approximate peak values of 100 pN vs. 200 pN respectively) and were also reduced after siRNA treatment of HEC-1B cells (approximate peak values of 200 pN vs. 100 pN; Fig. 3). Non-specific anti-GAPDH antibody functionalised probes were used as a negative control, demonstrating that binding events were specific to MUC1 (Fig. 3b). Furthermore, the number of positive binding events measured in HEC-1B cells decreased after siRNA treatment. This study reveals that mucins, and specifically MUC1, contributes to the intrinsic adhesive properties on the surface of endometrial cells.

### Development of an in vitro glycosylated MUC1 cell line model

**Fig. 4:**
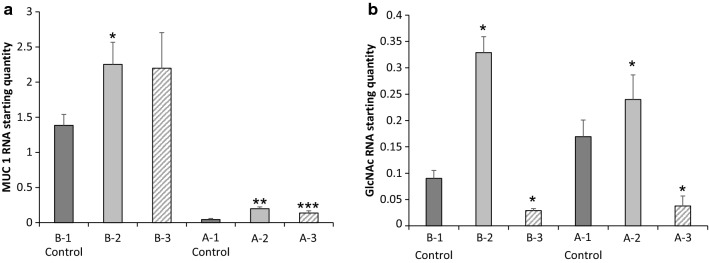
Mucin glycoprotein manipulation using inflammatory cytokine combinations.  **a** and **b** MUC1 and GLcNAc RNA expression data following treatment with TNFα and IFNγ cytokine treatment alone or in combination. (Models B-1 & A-1 – Control; B-2–200IU IFNγ + 25 ng/ml TNFα; B-3–25 ng/ml TNFα; A-2–200IU IFNγ; A-3–25 ng/ml TNFα). All qPCR data shown as average and SD of a minimum 3 repeats. Significance given as * p < 0.05, **p < 0.001, ***p < 0.0001

A series of cytokine-manipulated (TNFα and IFNγ) HEC-1A and HEC-1B cell line models were developed to elucidate the specific role of glycosylated MUC1 in mediating L-selectin binding, which is thought to be crucial in endometrial receptivity for the developing embryo (Fig. 4a and b). The highest MUC1 expression levels were again observed in HEC-1B cells (1.39 ± 0.15 to 2.25 ± 0.51; Additional file 1: Table S1) and were lowest in HEC-1A cells (0.05 ± 0.01 to 0.20 ± 0.02; Additional file 1: Table S1). The greatest increases in GlcNac expression occurred after treatment of the cell lines with IFNγ alone or in combination with TNFα (Model B-2–0.329 ± 0.030, Model A-2–0.240 ± 0.047; Additional file 1: Table S1), while a reduction in GlcNac expression occurred after treatment of the cell lines with TNFα alone (Model B-3–0.028 ± 0.003, Model A-3–0.038 ± 0.019, Additional file 1: Table S1). Importantly, the cytokine treatments did not alter the viability of either the HEC-1A and HEC-1B cells, as determined by MTT assays (Additional file 1: Fig. S1).

### Development of an in vitro L-selectin SMFS model

To elucidate the extrinsic adhesive properties of MUC1 in endometrial receptivity (Fig. 1a), we developed an *in vitro* SMFS assay by functionalising AFM probes with L-selectin. MUC1 has been implicated in fertility through the sialyl-Lewis X/L-selectin blastocyst adhesion system [4]. The functionalisation of AFM probes with L-selectin enabled the capacity for MUC1 to bind L-selectin to be characterised thereby providing mechanistic insights into blastocyst adherence at the endometrial surface. A flexible probe linker chemistry was used to ensure that L-selectin orientation was not limited, therefore maximising binding probability [28]. The presence of L-selectin on the AFM tip was inferred through comparing SMFS binding measurements on glass to those performed on HEC-1B and HEC-1A cell surfaces (Additional file 1: Fig. S2, Table S1). Non-specific anti-GAPDH antibody functionalised probes, as a negative control, demonstrated only background adhesion on live cell surfaces below 250 pN. Furthermore, L-selectin specific binding was validated through SMFS maps on HEC-1B and HEC-1A cells treated with MUC1 siRNA, showing a loss of binding (See Additional file 1: Fig. S2). The SMFS measurements made with the L-selectin functionalised probes revealed greater adhesion forces and adhesion energies for the MUC1-containing cell surfaces compared to the glass control (10.5 ± 0.9 vs. 325.9 ± 7 pN; 1.05 ± 0.27 vs. 15.9 ± 0.4 aJ) and also with force measurements using non-functionalised probes (9.03 ± 0.47 vs. 325.9 ± 7 pN; 0.59 ± 0.06 vs. 15.9 ± 0.4 aJ).

### Increase in MUC1 and GlcNac expression correlates with enhanced L-selectin binding

**Fig. 5:**
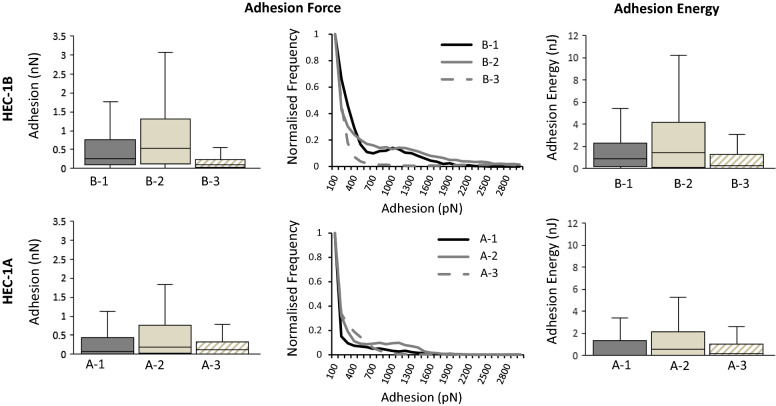
L-selectin adhesion to endometrial cell surface models. L-selectin binding to HEC-1A and HEC-1B cell models (B1-3 and A1-3) was characterized using a L-selectin functionalised AFM probe with SMFS, resulting in measurements of adhesion (nN) and adhesion energy (nJ). SMFS data shown as box plots and frequency histograms produced from a minimum of 8000 curves, 5 cells per sample and minimum of 3 biological repeats (n = 15)

In order to examine the relative contribution of glycosylated MUC1 in mediating L-selectin binding, HEC-1 cells were interrogated with the L-selectin functionalised AFM probes. The MUC1 rich HEC-1B cells demonstrated higher L-selectin mean binding values compared to the MUC1 poor HEC-1A cells for both adhesion force (0.466 ± 11 vs. 0.325 ± 7 nN) and binding distance (0.465 ± 4 vs. 0.343 ± 4 µm) (Fig. 5; Additional file 1: Table. S1 and  Fig. S3). Furthermore, despite the mean value of adhesion energies between the two cell lines being similar (1.8 ± 0.1 vs. 1.6 ± 0.04 nJ; Additional file 1: Table. S1), the median value was clearly higher for HEC-1B compared to HEC-1A cells (0.859 vs. 0.056 nJ; Fig. 5). Therefore, the MUC1 expression levels altered the magnitude of the L-selectin adhesive binding forces.

Secondly, the impact of GlcNAc expression in mediating L-selectin binding was assessed, where increased GlcNAc expression in both HEC-1B and HEC-1A (Models B-2 & A-2) resulted in increased adhesion force (1076 ± 18 and 428 ± 6 pN) and adhesion energy (7.1 ± 0.2 and 2.0 ± 0.05 nJ) (Fig. 5; Additional file 1: Table S1). The greatest increase was seen in HEC-1B cells which had the highest level of GlcNAc expression for all models (Model B-2) (Additional file 1: Table S1). Also, a reduction in GlcNAc expression in the HEC-1B and HEC-1A cell lines (Models B-3 & A-3) led to a decrease in adhesion force (352 ± 6 and 213 ± 4 pN) and adhesion energy (1.4 ± 0.02 and 0.8 ± 0.02 nJ) (Fig. 5). The greatest decrease in these binding measurements was seen in HEC-1A cells (Model A-3) even though the reduced level of GlcNAc expression was comparable to HEC-1B cells (Model B-3; Additional file 1: Table S1). These data suggest that both the presence of MUC1 and levels of glycosylation mediate the strength of L-selectin binding. Interestingly, quantification of MUC16 expression on the HEC-1 cell surface revealed that the MUC1 poor HEC-1A cells had increased expression of MUC16, whilst the MUC1 rich HEC-1B cells had reduced expression of MUC16 (Additional file 1: Fig S4). This data confirms the role of MUC1 in mediating the strength of L-selectin binding.

### Glycosylated MUC1 drives high affinity L-selectin binding events

**Fig. 6:**
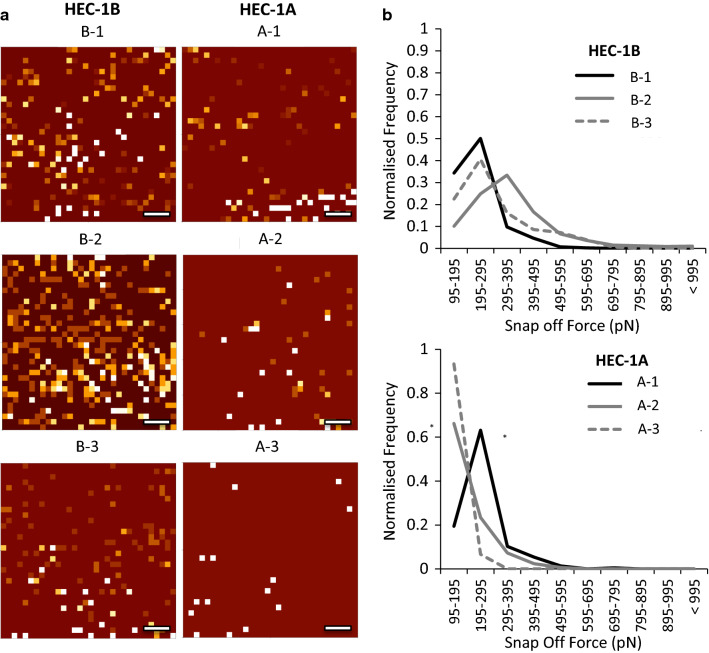
L-selectin binding events to endometrial cell surface models. **a** AFM SMFS force map images of individual L-selectin binding events across the HEC-1A and HEC-1B endometrial cell surface models (B1-3 and A1-3), scale bar 1.5 µm. **b** Corresponding L-selectin binding event data related to the snap-off forces (pN) of the functionalized probe from the HEC-1A and HEC-1B cell models. All SMFS data shown in B is total number of events across a minimum of 6000 curves, across 5 cells, from 3 biological repeats (n = 15) [40]

To assess the frequency of L-selectin binding events in relation to the MUC1 and glycosylation expression, force curve binding event mapping was performed (Fig. 6a). The VNTR structure of MUC1 is highly glycosylated and decorated with sialyl-Lewis X and therefore acts as a scaffold for interactions with L-selectin [24, 29]. A greater number of L-selectin binding events were detected in MUC1 rich models (B-1 to B-3) compared to the MUC1 poor models (A-1 to A-3), which suggests that frequency of L-selectin binding increases when MUC1 is in abundance. Also, increased glycosylation in MUC1 rich models (B-2) further enhanced the frequency of L-selectin binding events.

The tandemly repeating sequences within the VNTR structure of MUC1 could also potentially facilitate multiple interactions between the L-selectin functionalised probe and a single MUC1 molecule [24, 29]. Measurement of the snap off forces produced during the AFM probe retraction can reveal the strength of individual bonds made between the L-selectin and MUC1 molecules (Additional file 1: Fig. S5a). The snap off forces measured for L-selectin binding to cell surfaces revealed that when expression of MUC1 and GlcNAc was high (Model B-2) the frequency of high affinity binding events increased to 400-600 pN (Fig. 6b, Additional file 1: Fig. S5b-g). When MUC1 expression was low but GlcNAc expression was high (Model A-2), the frequency of high affinity binding events was reduced to 200-400pN. The frequency of L-selectin binding events was further reduced when both MUC1 and GlcNAc expression was low, where 90 % of L-selectin binding events have snap off forces below 195pN. These data demonstrate that highly glycosylated MUC1 drives the high affinity binding forces of L-selectin at the cell surface.

## Discussion

The lack of clinically relevant model systems describing human fertility has resulted in a limited understanding of the functional roles of key cell surface molecules during implantation. Mucins present on the surface of endometrial epithelial, such as MUC1, have been implicated in fertility through a sialyl-Lewis X/L-selectin blastocyst adhesion system [13, 14]. However, the adhesive and anti-adhesive properties of these mucins at the endometrial cell surface is difficult to resolve due to known discrepancies between animal models and the human reproductive system, and also a lack of sensitivity in current *in vitro* models, meaning the role of MUC1 mucins in the reproductive process had remained elusive [13–15, 17, 18]. To overcome these difficulties, we developed an *in vitro* approach using AFM analysis to not only decipher the intrinsic and extrinsic roles of MUC1 but also to examine the role of glycosylation in L-selectin binding.

AFM force measurements have been widely used to compare the mechanical properties of physiological and pathological cells within fertility and cancer research [30, 31]. Hsu et al. 2016, measured the nanomechanical properties of RL95-2 cells (an endometrial carcinoma cell line) using PF-QNM where elasticity values ranged between 1 and 35 kPa and the adhesion between 200 and 1900 pN, [32] which correlates well with nanomechanical measurements obtained from the HEC-1 cells used in this study. Other researchers have examined the elasticity of Ishikawa (endometrial adenocarcinoma), HeLa (cervix adenocarcinoma) and MCF (breast adenocarcinoma) cells where the elasticity ranges from 0.7 to 2.73 kPa [33, 34]. Such measurements were obtained with a colloidal AFM probe and in ‘force mapping’ mode, rather than PF-QNM mode, which will have contributed to lower values of elasticity measured.

To examine if high intrinsic adhesion forces obtained at the cell surface were linked to MUC1 expression, the surface of the endometrial HEC-1A and HEC-1B cell lines were examined with AFM, and MUC1 siRNA ablation assays revealed a correlation between reduced MUC1 expression and a reduction in adhesion forces. Furthermore, anti-MUC1 antibody functionalised AFM probes used in SMFS anaysis revealed that increased MUC1 expression led to an increase in the magnitude of rupture forces at the cell surface, which reduced with siRNA MUC1 ablation. Sulchek et al., 2005, demonstrated that the force required for MUC1-antibody bond separation is directly proportional to the number of bonds [35], where the rupture force for a single bond between a single-chain variable fragment (scFv) fusion MUC1 antibody and a MUC1 peptide was approximately 150 pN. In this study, the most frequent rupture force measured from MUC1 poor HEC-1A cells was between 100 and 200 pN, which is equivalent to a single MUC1-mAb interaction. In contrast MUC1 rich HEC-1B cell rupture forces generally occurred in the 200–300 pN range, suggesting that this rupture force resulted from multiple bond separations [36], possibly due to increased expression of MUC1 on the cell surface. The presence of larger rupture forces occurring at relatively long distances away from the cell surface (see Additional file 1: Figure S4b-g) implies that the probe is breaking multiple bonds on the MUC1 molecule (specific adhesion molecules on the cell surface do not extend beyond > 50nm). These assays suggest that MUC1 contributes to intrinsic adhesive properties when present on the human endometrial cell surface.

Cytokines are involved in menstruation and implantation processes within the endometrium [37], where cytokine expression reaches a maximum in the mid-secretory phase of the menstruation cycle, concurrent with the window of implantation [38]. Interestingly, proinflammatory cytokines TNFα and IFNγ are present within the human endometrium throughout the menstrual cycle. TNFα is expressed by the human endometrium in response to the presence of steroid hormones and can be detected in maternal sera and conditioned medium of preimplantation blastocysts, while TNFα receptors, TNFR-I and TNFR-II, are found expressed in the endometrial epithelium and in blastocysts. IFNγ is expressed in the luminal and glandular epithelia and IFNγ receptors, IFNγR-1 and IFNγR-2, are found expressed in the human endometrium [39]. Studies have found that expression of high levels of TNFα and IFNγ are implicated in pregnancy loss in *in vivo* studies [39]. These proinflammatory cytokines greatly stimulate MUC1 expression in various cellular models through STAT1α and nuclear factor B (NFкB) transcription factors binding to STAT and кB within in the MUC1 promoter [40–42]. Therefore, TNFα and IFNγ are biologically relevant treatments to stimulate MUC1 expression.

In our study, we used the HEC-1A and HEC-1B cell lines treated with TNFα and IFNγ to model possible fertile/infertile pathologies. The development of this *in vitro* model allowed us to not only compare MUC1 rich systems to MUC1 poor systems but also to compare varying levels of glycosylation, making the model extremely useful in deciphering the role of glycosylated MUC1 in fertility. Interestingly, when IFNγ was used alone or in combination with TNFα, there was an increase in mucin glycosylation in both cell lines, however, when TNFα was used alone there was a decrease in mucin glycosylation. The variation in mucin expression and glycosylation within our models could be linked to the dysregulated cytokine release in infertile patients, suggesting that cytokine interaction with their receptors may indirectly effect blastocyst apposition and adhesion to the endometrium surface [38].

Interestingly, studies have been performed using MECA-79, an antibody that recognises 6-sulfo sialyl-Lewis X [43], and demonstrated that MECA-79 epitope selectin ligands increased from the proliferative to the secretory phase of the menstrual cycle in fertile individuals [29, 44], whilst the lack of MECA-79 expression in mid-luteal endometrial biopsies revealed a low or no chance of pregnancy [45]. Carson et al., 2006 demonstrated MECA-79 binds to MUC1 indicating the presence of sialyl-Lewis X on the glycosylated mucins [19] and Margarit et al. 2009 correlated decreased expression of GlcNAc6ST-2 in fertile patients with decreased MECA-79 expression [20]. These studies reveal the importance of sialyl-Lewis X and glycosylation in human fertility, however an *in vitro* model to fully characterise the interaction between sialyl-Lewis X and its ligand L-selectin has been lacking. Our study has developed a reliable SMFS *in vitro* model capable of quantifying the extrinsic adhesive interactions between L-selectin and sialyl-Lewis X carrying mucins which are implicated in blastocyst binding to the endometrial cell surface.

The initial interaction of the blastocyst with the cell surface occurs with the transmembrane mucins which extend well beyond the cell surface (200-500 nm) where only MUC1 and MUC16 molecules are present due to their extended structures [46, 47]. To elucidate the extrinsic adhesion interactions occurring between L-selectin and glycosylated mucins, SMFS was used characterise this selectin-ligand interaction [26, 48], and revealed that L-selectin binds more strongly to the ligands present on MUC1 rich cell surfaces when compared to MUC1 poor cell surfaces. Furthermore, L-selectin appeared to bind more strongly to highly glycosylated MUC1, indicating that blastocyst adhesion to highly glycosylated MUC1 mucins would be enhanced. Cellular AFM force mapping combined with examination of the snap-off forces provided further confirmation of the importance of highly glycosylated MUC1 in fertility, as L-selectin binding events were greater in frequency and magnitude (400–600 pN) on highly glycosylated MUC1 rich cell lines compared to highly glycosylated MUC1 poor cell lines (200–400 pN). As the force required for bond separation is directly proportional to the number of bonds [24], the increase in the magnitude of L-selectin binding for highly glycosylated MUC1 is potentially due to multiple bonds being formed between the L-selectin functionalised probe and the VNTR on the mucin strands. Importantly, this data demonstrates the significance of highly glycosylated MUC1 in the adhesion mechanism to L-selectin. Other cell surface adhesion related molecules do not extend beyond 50 nm from the cell surface [46, 47]. Therefore, the long-range interactions that occur between the mucin molecules and the AFM probe can be successful captured by AFM Force Retraction experiments.

## Conclusions

The ability of the blastocyst to implant into the endometrial surface is a delicate balance between the adhesive and anti-adhesive properties of the mucin protein dominated endometrial surface layer [15–17]. Previous *in vivo* studies have demonstrated that the mucin layer acts as a natural barrier to the embryo attachment however, human expression of MUC1 is high during per-implantation [17, 18]. This study demonstrated that MUC1 contributes to both the intrinsic and extrinsic cellular adhesive properties which may function to facilitate trophoblast binding to the endometrial cell surface through the L-selectin/sialyl-Lewis x adhesion system before subsequent implantation. MUC1 extrinsic adhesive properties are linked to mucin glycosylation status, where greater glycosylation results in increased L-selectin binding. The heterogeneity of MUC1 expression in the endometrium may define a receptive site for implantation, facilitating the initial adhesion of blastocyst to the endometrial surface layer before implantation [17]. Furthermore, the application of SMFS is a unique approach in the endometrium which has been able to quantify the strength and frequency of L-selectin binding and determine the functional availability of mucins at the nano-meter resolution [49–51].

## Methods

### Cell culture

Endometrial adenocarcinoma HEC-1A and HEC-1B cell lines (ATCC® HTB-112 and ATCC® HTB-113 respectively; www.lgcstandards-atcc.org) [52] were maintained (37^o^C, 5 % CO_2_) in DMEM/F-12 + Glutamax™ full media (Gibco, Thermo Fisher, UK) supplemented with 10 % (v/v) foetal bovine serum (FBS), sodium bicarbonate 1mM, sodium pyruvate 1mM and 1 % (v/v) antibiotic-antimycotic solution in plastic culture vessels (25 cm^2^, 75 cm^2^, 125 cm^2^). Cells were supplemented with full serum media every two days and passaged when confluent. Only cells passaged more than two times were used in this study.

### Atomic force microscopy PFQNM imaging

Cell lines were cultured in 50 mm diameter glass-bottomed dishes (WillCo Wells, Amsterdam, The Netherlands) for use on the Bioscope Catalyst II AFM (Bruker, Coventry, UK). After the desired treatment regime, the culture media was replaced with 2 ml of pre-warmed DMEM/F-12 phenol red free media (Gibco, Thermo Fisher, UK) prior to live cell imaging. Cells were imaged over a maximum time of 90 mins at 37 °C. ScanAsyst Fluid cantilevers (Bruker, Coventry, UK) were used with a nominal spring constant of 0.7 N/m and a tip apex radius of 20–60 nm. The indentation force was kept below < 1 nN, scan rate was 0.5 Hz, resolution was 128 samples/line and scan area encompassed 50–150 µm of the cell monolayer. Processing of PFQNM images was undertaken with the Bruker Nanoscope software, following standard protocol [53, 54]. Data shown is based on a minimum of three biological repeats, analysed as parametric data using 2 tailed T test. Significance given as * p < 0.05, ** p < 0.001 and *** p < 0.0001.

### MUC1 siRNA preparation

HEC-1A and HEC-1B cells were removed from the culture vessels with 0.25 % (w/v) trypsin-EDTA (Gibco, Thermo Fisher, UK) and re-suspended in cell culture media. 1 ml of cell suspension (approximately 8 × 10^4^ cells) was aspirated onto round glass-bottomed 30 mm dishes (WillCo Wells, Amsterdam, The Netherlands), and cells allowed to attach for 5 mins. 2 ml of cell culture media was then added and cells incubated for 24 h (37 °C, 5 % CO_2_). The cell culture medium was then replaced with 2.5 ml antibiotic free culture media supplemented with 500 µl OPTI-C medium, 10 µl MUC1 siRNA (Santa Cruz Biotechnology, Heidelberg, Germany) and 7.5 µl lipofectamine RNAiMAX (Invitrogen) before the cells were further incubated for 48 h (37 °C, 5 % CO_2_). This transfection preparation was incubated for 20 mins at 25 °C prior to application.

### Population based MUC1 protein screening

Total protein was assessed with IN Cell analyser 2000 (GE Healthcare, Amersham, UK). Cells were fixed with 4 % (w/v) paraformaldehyde then stained with mouse MUC1 ND antibody (Santa Cruz Biotechnology, Heidelberg, Germany) prior to incubation with an anti-mouse Texas Red conjugated secondary antibody (emission 620 nm; Molecular Probes, Thermo Fisher UK). Cell nuclei were counter stained with 4’,6-diamidino-2-phenylindole, DAPI (emission 470 nm; Invitrogen, Thermo Fisher, UK), where PBS wash steps followed each staining process. Five low magnification images of approximately 1000 cells in total were achieved. An object segmentation protocol within the IN Cell Developer software masked the nuclei by segmenting on intensity in the DAPI channel. Information about the fluorescence output in the Texas Red channel (MUC1) within this ‘cell region’ was recorded and each cell assigned an output value. All data shown is based on a minimum of three biological repeats, analysed as parametric data using 2 tailed T test. Significance given as * p < 0.05, **p < 0.001.

### Functionalization (anti-MUC1 antibody, anti-GAPDH antibody and L-selectin) of AFM probes

Direct functionalization of AFM probes (DNP-10 D tips, Bruker, Coventry, UK) was performed by rinsing the probes in deionised H_2_O x5, before immersion in HAPTES buffer (0.1 % v/v, pH 7.0; Sigma Aldrich, Gillingham, UK) for 7 mins at 25 °C. The sialianized probes were rinsed x5 in deionised H_2_O before immersion in glutaraldehyde (0.5 % w/v pH 7.0) for 7 mins at 25 °C. The probes were further rinsed in deionised H_2_O before being immersed in either 200 µg/ml mouse VU4H5 anti-MUC1 antibody or 200 µg/ml mouse anti-GAPDH antibody (Santa Cruz Biotechnology, Heidelberg, Germany) for 15 mins at 25 °C. The AFM probes were then rinsed x5 in Tris HCl (5 % w/v pH 7.0) before immersion and storage in Tris HCl. Linker functionalisation of AFM probes with L-selectin and associated negative control followed a two-step chemical procedure [28]. The AFM probes (DNP-10; Bruker-nano, Coventry, UK) were briefly washed in acetone for 5 mins before immersion in piranha solution (H_2_SO_4_:H_2_O_2_; 3:1; v/v) for 30 mins. The cantilevers were then incubated in 1 mL of APTES (0.1 % w/v, pH 7.2) for 10 mins to create an amino-terminated tip surface. The probes were then rinsed with PBS (x5) followed by rinsing with water (x5) before incubation in LC-SPDP (succinimidyl 6-(3(2-pyrifyldithio)propionamido)hexanoate) for 45 mins to obtain a reactive pyridyl-disulfide surface. The LC-SPDP functionalised probes were then rinsed again in PBS (x5) and water (x5). L-selectin (200 µg/mL; Randox Laboratories, County Antrim, UK) was modified by reaction with SATP (N-succinimidyl-S-acetylthiopropionate) for 30 mins in order to produce a free sulfhydryl group, before a series of purification steps through a dextran salting column (5.0k MWCO; Pierce^™^, Thermo Fisher, UK) were performed. LC-SPDP functionalized probes were then incubated in the presence of thiolated-activated L-selectin, forming an L-selectin functionalised probe through a disulfide exchange reaction with SPDP-activated protein [55]. The AFM probes were functionalised fresh before every experiment and kept submerged in a solution of 20 mM sodium phosphate, 0.15 M NaCl and 10 mM EDTA at pH 7.2. ATPES was freshly made and adjusted to pH 7 before the functionalisation to prevent hydrolyses of ATPES which could interfere with sialinization reaction with the AFM tip.

### Force mapping with the functionalised probe

Cell lines were cultured in 35 mm diameter glass-bottomed dishes (Fluorodish, WPI Precision Instruments, Hertforshire, UK) for use on the Nanowizard II AFM (JPK BioAFM, Berlin, Germany). HEC-1A and HEC-1B cell monolayers were probed for single molecule force adhesive interactions by using L-selectin functionalised, anti-GAPDH functionalised and anti-MUC1 antibody functionalised AFM probes at 37 °C (DNP-10 D tips, Bruker Nano, Coventry, UK). AFM probes functionalised with anti-GAPDH were used as a negative control as the cell surface is devoid of any GAPDH. Probes with nominal spring constants of 0.065 N/m following functionalization were used (confirmed with the Thermal tune method). The maximum load force was 1.5 nN, z speed was 3 µm/s (extend and retract) and a force delay of 200 ms applied. Force volume maps with 16–32 pixels/side were acquired on a minimum of 3 cellular locations, where each experiment was performed at least three times with different yet identically prepared AFM probes and surfaces. This resulted in a minimum of 768 force curves per sample type. Moderate variations in the noise of force curves across measurements are due to variations in the AFM probes.

All pixels/points for each force curve (1024) was collected and included in the retraction curve data to ensure all binding events were considered. All force curve quality control features of the JPK software were used. Only curves where the retraction curve returned to the baseline were included for analysis and a goodness of fit of R = 0.85 or more were used. Vertical deflection (baseline) and contact point were adjusted to 0 nm. Identical processing conditions were applied to all force volume files using a saved batch processing algorithm.

Force curves were analyzed with JPK data processing software to fit steps to the retraction curves in order to detect specific surface interactions. Steps were fitted to the curve using in built features of the software providing data on the size of the step (pN) and the distance it occurred from the contact point. Only rupture events appearing at tip-sample distances larger than 200 nm were considered for further analysis to avoid bias by nonspecific tip-sample interactions. The two parameters to control the algorithm are “smoothing’’ which defines how smooth the ‘slowly varying background signal’ is and ‘significance’ which sets the threshold below which steps are considered to be noise (and are thus discarded). A significance value of 0.001 means that the only step heights accepted have a probability of less than 1/1000 of being due to noise fluctuations (based on an estimate for the RMS of F_noise) .

The SMFS force maps with L-selectin functionalised probes illustrate the spatial location of each binding occurrence on the cell surface and the colour of the pixel indicates the number of binding steps experienced on the retraction curve. For the MUC1-antibody functionalised probes, the frequency of surface binding was displayed as the percentage of positive and negative binding force curves where a student’s 2 tailed T-test was performed to assess significance. Significance given as *p < 0.05.

### Cytokine treatment

Prior to cytokine treatment, cell culture media containing stripped FBS serum was added to the HEC-1A and HEC-1B cell cultures for 24 h to ensure the removal of large molecular weight proteins and steroids. The cells were then treated with two cytokines TNFα and IFNγ (Miltenyi Biotec, Woking, UK) either alone or in combination for 48 h (37 °C, 5 % CO_2_). HEC-1B and HEC-1A cell models (B-1 and A-1) were the control samples. In the HEC-1B cell line, model B-2 consisted of the treatment 200 IU IFNγ + 25 ng/ml TNFα while model B-3 consisted of the treatment 25 ng/ml TNFα. In the HEC-1A cell line, model A-2 consisted of the treatment 200IU IFNγ, while model A-3 used the treatment 25 ng/ml TNFα.

### Real time quantitative polymerase chain reaction (RT-QPCR)

Total RNA was isolated from the cell models using the RNeasy mini and DNAseI kit (74,106 and 79,254; Qiagen, Manchester, UK) following the manufacturer’s instructions. The concentration of extracted total RNA was measured using a Nano-drop ND-1000 spectrophotometer (Thermo Fisher, UK) and samples adjusted to 100 µg/ml RNA using RNAse free H_2_0 (Qiagen, Manchester, UK). cDNA was obtained using the high capacity cDNA kit (4,387,406, Applied Biosystems, Thermo Fisher, UK) and amplified using gene specific primer pairs to obtain a PCR product between 75 and 263 bp for each of the genes under study (MUC1, GlcNac, see Additional file 2). Real time QPCR amplifications were conducted in triplicate in 96-well optical reaction plates (BioRad, Watford, UK) and run on the CfX-96 PCR detection system (Biorad, Watford, UK). RP-L19 was used as a housekeeping gene, and genomic DNA and RNA were used as positive and negative controls respectively. All expression levels were normalized with values obtained for the internal reference gene RP-L19. Data shown was analysed as using one-way ANOVA test. Significance given as *p < 0.05.

## Supplementary Information

**Additional file 1: Table S1.** Molecular determinants of L-selectin affinty to the endometrial HEC-1A and HEC-1B cell surface model. **Figure S1.** Stable cell viability in the presence of cytokine mediated alterations in mucin glycoprotein expression. *Figure S2.* SMFS control experiments. **Figure S3.** L-selectin binding to HEC-1A and HEC-1B endometrial cell surface models resulting in measurements of distance (μm). **Figure S4.** MUC16 glycoprotein manipulation using inflammatory cytokine combinations. **Figure S5.** SMFS force distance curve analysis methods and example force curves achieved from L-selectin functionalized AFM probes to HEC-1A and HEC-1B cells.

**Additional file 2.** Real time Quantitative Polymerase Chain Reaction primer sequences.

## Data Availability

The datasets used and/or analysed during the current study are available from the corresponding author on reasonable request.
