# Supplementary material for: Highly glycosylated MUC1 mediates high affinity L-selectin binding at the human endometrial surface
Source: J Nanobiotechnology. 2021 Feb 17;19:50. doi: 10.1186/s12951-021-00793-9 (PMC7890821; doi:10.1186/s12951-021-00793-9)
Supplement: Supplementary file 1 — Additional file 1: Table S1. Molecular determinants of L-selectin affinty to the endometrial HEC-1A and HEC-1B cell surface model. Figure S1. Stable cell viability in the presence of cytokine mediated alterations in mucin glycoprotein expression. Figure S2. SMFS control experiments. Figure S3. L-selectin binding to HEC-1A and HEC-1B endometrial cell surface models resulting in measurements of distance (μm). Figure S4. MUC16 glycoprotein manipulation using inflammatory cytokine combinations. Figure S5. SMFS force distance curve analysis methods and example force curves achieved from L-selectin functionalized AFM probes to HEC-1A and HEC-1B cells. [file 12951_2021_793_MOESM1_ESM.pptx]

## Slide 1
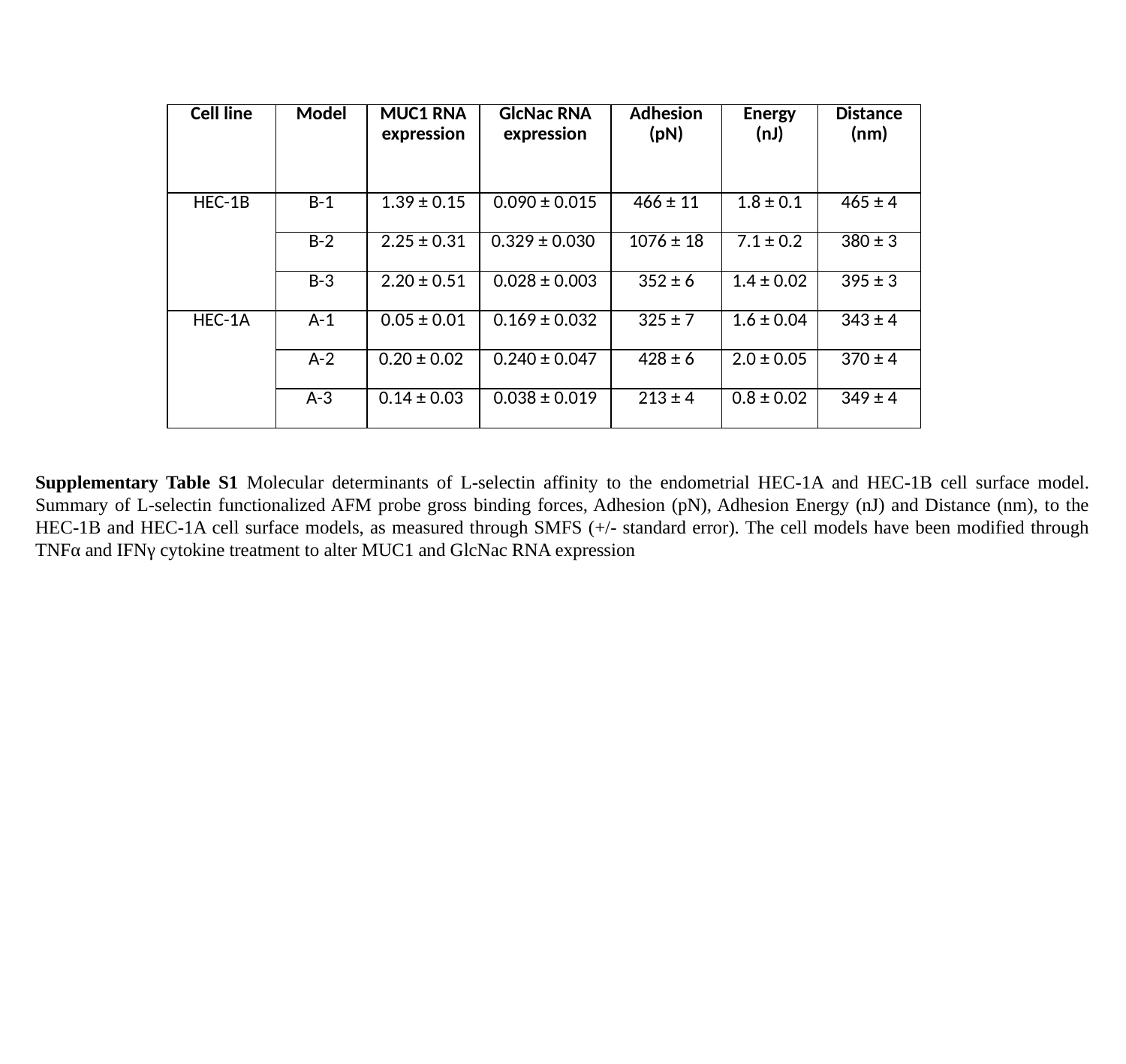

| Cell line | Model | MUC1 RNA expression | GlcNac RNA expression | Adhesion (pN) | Energy (nJ) | Distance (nm) |
| --- | --- | --- | --- | --- | --- | --- |
| HEC-1B | B-1 | 1.39 ± 0.15 | 0.090 ± 0.015 | 466 ± 11 | 1.8 ± 0.1 | 465 ± 4 |
| | B-2 | 2.25 ± 0.31 | 0.329 ± 0.030 | 1076 ± 18 | 7.1 ± 0.2 | 380 ± 3 |
| | B-3 | 2.20 ± 0.51 | 0.028 ± 0.003 | 352 ± 6 | 1.4 ± 0.02 | 395 ± 3 |
| HEC-1A | A-1 | 0.05 ± 0.01 | 0.169 ± 0.032 | 325 ± 7 | 1.6 ± 0.04 | 343 ± 4 |
| | A-2 | 0.20 ± 0.02 | 0.240 ± 0.047 | 428 ± 6 | 2.0 ± 0.05 | 370 ± 4 |
| | A-3 | 0.14 ± 0.03 | 0.038 ± 0.019 | 213 ± 4 | 0.8 ± 0.02 | 349 ± 4 |
Supplementary Table S1 Molecular determinants of L-selectin affinity to the endometrial HEC-1A and HEC-1B cell surface model. Summary of L-selectin functionalized AFM probe gross binding forces, Adhesion (pN), Adhesion Energy (nJ) and Distance (nm), to the HEC-1B and HEC-1A cell surface models, as measured through SMFS (+/- standard error). The cell models have been modified through TNFα and IFNγ cytokine treatment to alter MUC1 and GlcNac RNA expression

## Slide 2
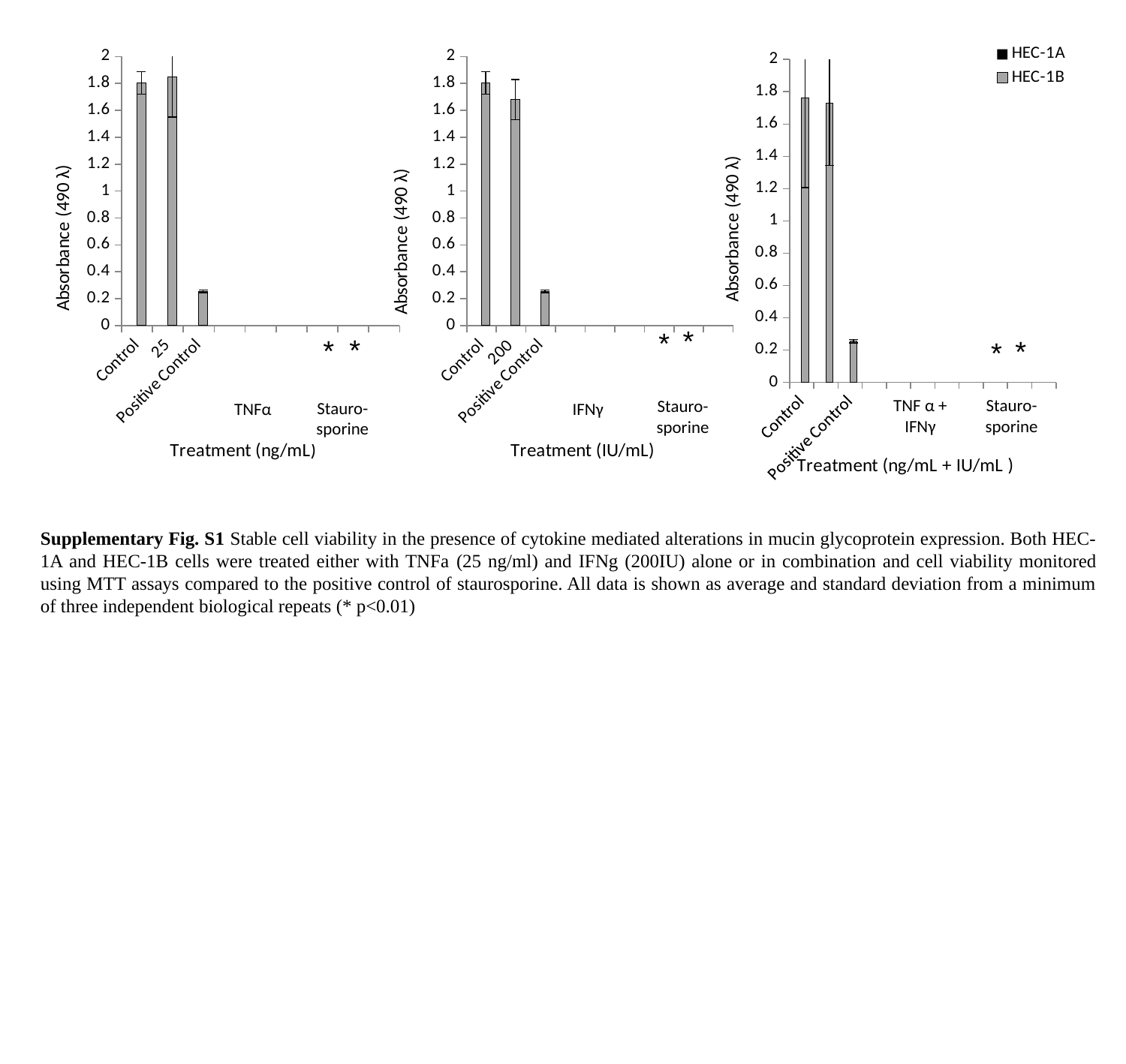

### Chart
| Category | HEC-1A | HEC-1B |
|---|---|---|
| Control | 1.1734166666666668 | 1.7594166666666664 |
| 200 + 25 | 1.1806666666666668 | 1.7294999999999998 |
| Positive Control | 0.24200000000000002 | 0.25416666666666665 |
### Chart
| Category | | HEC-1B |
|---|---|---|
| Control | 1.4371666666666667 | 1.8038333333333334 |
| 25 | 1.2755 | 1.8483333333333334 |
| Positive Control | 0.24200000000000002 | 0.25416666666666665 |
### Chart
| Category | HEC-1A | HEC-1B |
|---|---|---|
| Control | 1.4371666666666667 | 1.8038333333333334 |
| 200 | 1.1386666666666665 | 1.6801666666666668 |
| Positive Control | 0.24200000000000002 | 0.25416666666666665 |*
*
*
*
*
*
Stauro-sporine
TNF α + IFNγ
Stauro-sporine
Stauro-sporine
TNFα
IFNγ
Supplementary Fig. S1 Stable cell viability in the presence of cytokine mediated alterations in mucin glycoprotein expression. Both HEC-1A and HEC-1B cells were treated either with TNFa (25 ng/ml) and IFNg (200IU) alone or in combination and cell viability monitored using MTT assays compared to the positive control of staurosporine. All data is shown as average and standard deviation from a minimum of three independent biological repeats (* p<0.01)

## Slide 3
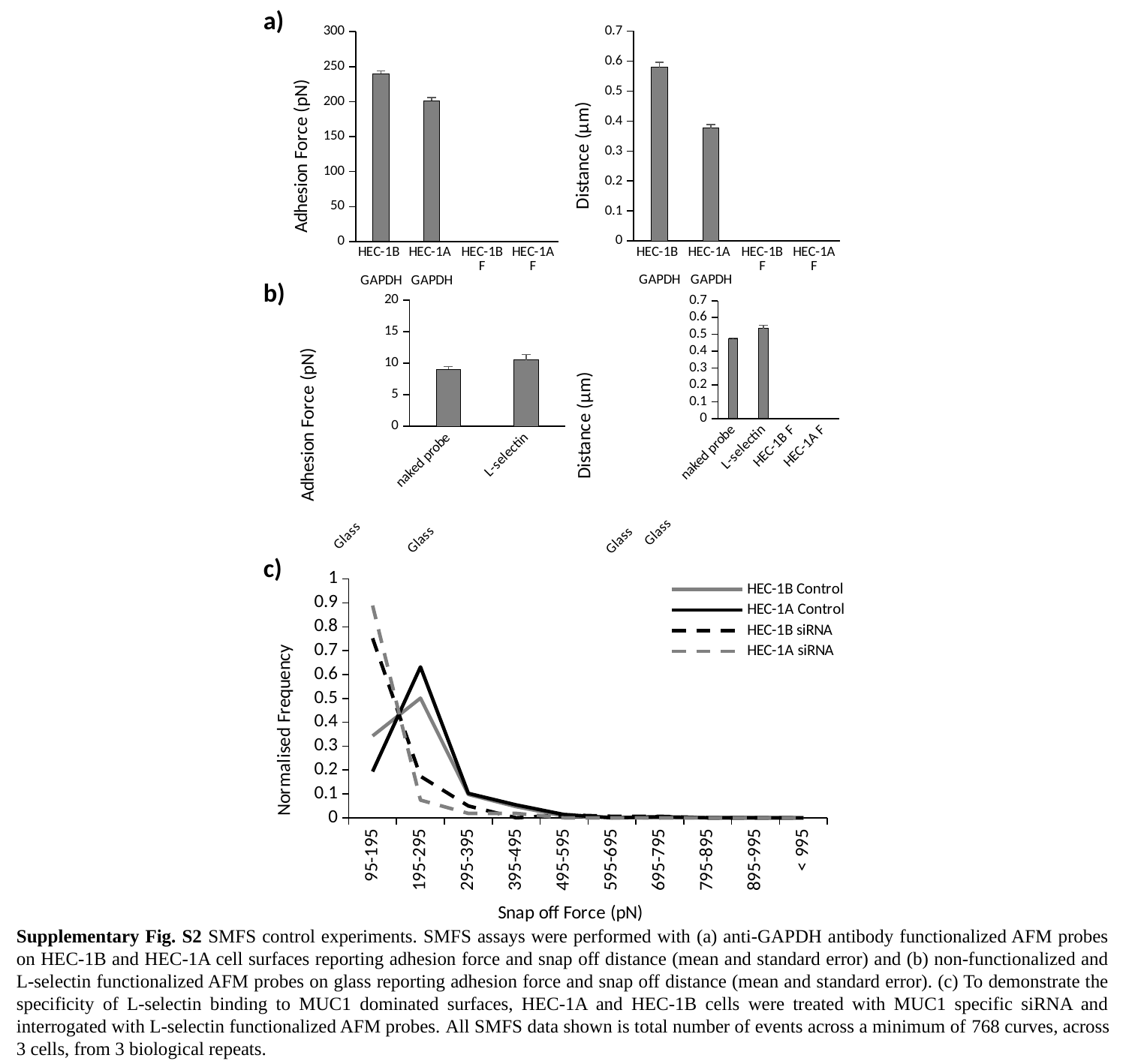

a)
[unsupported chart]
[unsupported chart]
b)
### Chart
| Category | |
|---|---|
| Glass naked probe | 9.02876 |
| Glass L-selectin | 10.5201 |
[unsupported chart]
c)
### Chart
| Category | | | | |
|---|---|---|---|---|
| 95-195 | 0.34288747346072185 | 0.19378427787934185 | 0.7515527950310559 | 0.8888888888888888 |
| 195-295 | 0.5010615711252654 | 0.6316270566727605 | 0.17391304347826086 | 0.07407407407407407 |
| 295-395 | 0.09766454352441614 | 0.10237659963436929 | 0.049689440993788817 | 0.018518518518518517 |
| 395-495 | 0.04670912951167728 | 0.05393053016453382 | 0.0 | 0.018518518518518517 |
| 495-595 | 0.0074309978768577496 | 0.013711151736745886 | 0.012422360248447204 | 0.0 |
| 595-695 | 0.0021231422505307855 | 0.0 | 0.006211180124223602 | 0.0 |
| 695-795 | 0.0 | 0.004570383912248629 | 0.006211180124223602 | 0.0 |
| 795-895 | 0.0010615711252653928 | 0.0 | 0.0 | 0.0 |
| 895-995 | 0.0010615711252653928 | 0.0 | 0.0 | 0.0 |
| < 995 | 0.0 | 0.0 | 0.0 | 0.0 |Supplementary Fig. S2 SMFS control experiments. SMFS assays were performed with (a) anti-GAPDH antibody functionalized AFM probes on HEC-1B and HEC-1A cell surfaces reporting adhesion force and snap off distance (mean and standard error) and (b) non-functionalized and L-selectin functionalized AFM probes on glass reporting adhesion force and snap off distance (mean and standard error). (c) To demonstrate the specificity of L-selectin binding to MUC1 dominated surfaces, HEC-1A and HEC-1B cells were treated with MUC1 specific siRNA and interrogated with L-selectin functionalized AFM probes. All SMFS data shown is total number of events across a minimum of 768 curves, across 3 cells, from 3 biological repeats.

## Slide 4
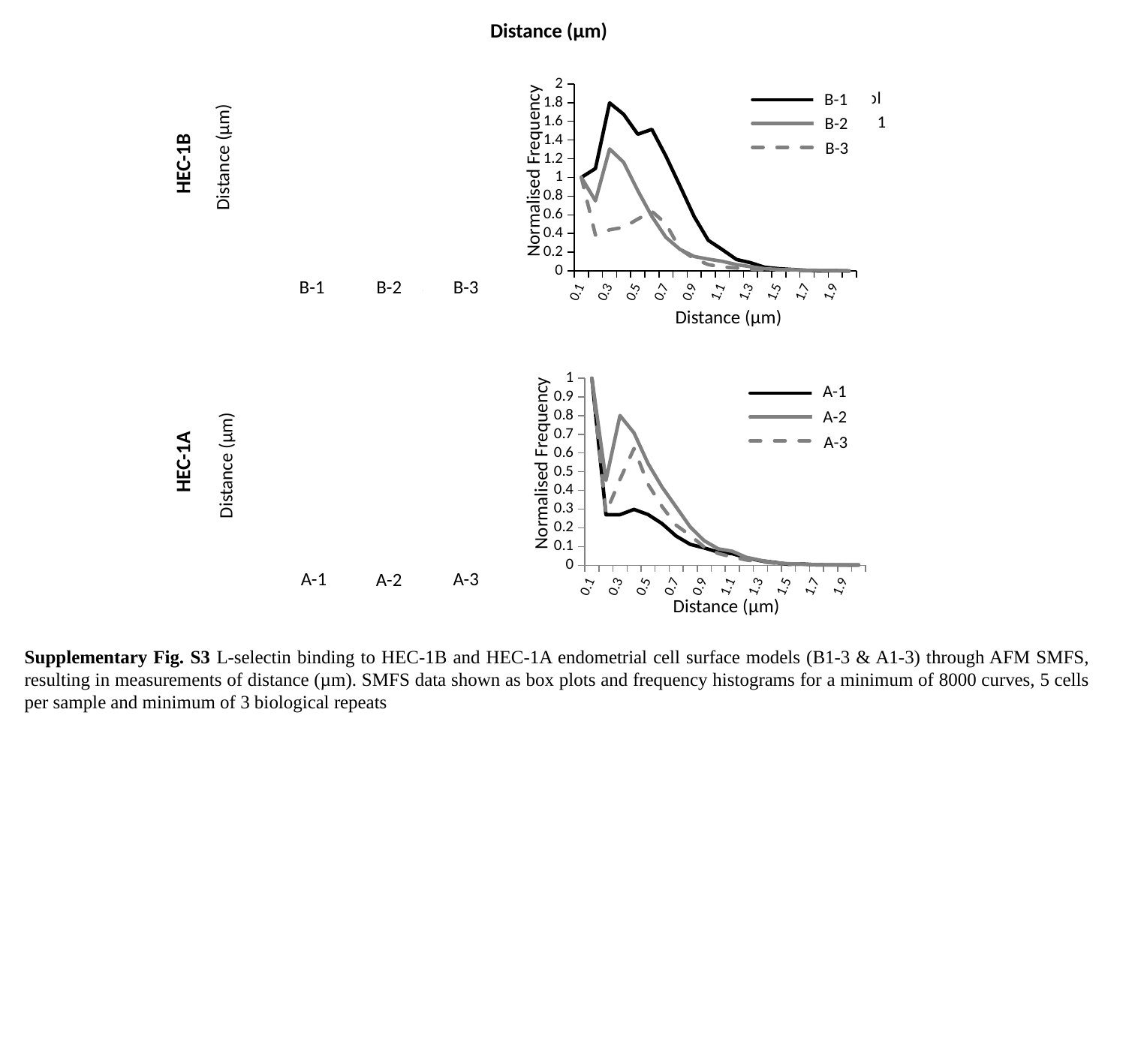

Distance (µm)
Distance (µm)
Model 2
Control
Model 1
Distance (µm)
Model 3
Control
Model 4
### Chart
| Category | | | |
|---|---|---|---|
| 0.1 | 1.0 | 1.0 | 1.0 |
| 0.2 | 1.0953545232273838 | 0.7501933488012374 | 0.38386090057958094 |
| 0.30000000000000004 | 1.7970660146699267 | 1.3047177107501933 | 0.439589835042354 |
| 0.4 | 1.6748166259168704 | 1.1616395978344933 | 0.46678555506018726 |
| 0.5 | 1.4621026894865525 | 0.8592420726991493 | 0.5541685242978154 |
| 0.6 | 1.5134474327628362 | 0.5831399845320959 | 0.637539010254124 |
| 0.7 | 1.2273838630806846 | 0.3588553750966744 | 0.5046812304948729 |
| 0.79999999999999993 | 0.9070904645476773 | 0.23047177107501934 | 0.23316986179224253 |
| 0.89999999999999991 | 0.5819070904645477 | 0.15467904098994587 | 0.12973695942933572 |
| 0.99999999999999989 | 0.3276283618581907 | 0.12606341840680588 | 0.06776638430673206 |
| 1.0999999999999999 | 0.2273838630806846 | 0.102861562258314 | 0.04190815871600535 |
| 1.2 | 0.12224938875305623 | 0.06728538283062645 | 0.03120820329915292 |
| 1.3 | 0.08801955990220049 | 0.04563031709203403 | 0.018279090503789567 |
| 1.4000000000000001 | 0.039119804400977995 | 0.02397525135344161 | 0.012483281319661168 |
| 1.5000000000000002 | 0.02444987775061125 | 0.016241299303944315 | 0.01069995541685243 |
| 1.6000000000000003 | 0.014669926650366748 | 0.01237432327919567 | 0.007579135086937138 |
| 1.7000000000000004 | 0.004889975550122249 | 0.007733952049497293 | 0.002229157378510923 |
| 1.8000000000000005 | 0.0 | 0.005413766434648105 | 0.0026749888542131075 |
| 1.9000000000000006 | 0.0024449877750611247 | 0.0038669760247486465 | 0.002229157378510923 |
| 2.0000000000000004 | 0.0 | 0.0 | 0.0008916629514043691 |Normalised Frequency
Distance (µm)
### Chart
| Category | | | |
|---|---|---|---|
| 0.1 | 1.0 | 1.0 | 1.0 |
| 0.2 | 0.26968605249613997 | 0.45354791514264814 | 0.2800480769230769 |
| 0.30000000000000004 | 0.27020072053525473 | 0.8002926115581566 | 0.4579326923076923 |
| 0.4 | 0.29850746268656714 | 0.7073884418434528 | 0.6243990384615384 |
| 0.5 | 0.27071538857436955 | 0.5435259692757864 | 0.4326923076923077 |
| 0.6 | 0.22233659289758106 | 0.4177029992684711 | 0.3137019230769231 |
| 0.7 | 0.1559444158517756 | 0.31163130943672274 | 0.21394230769230768 |
| 0.79999999999999993 | 0.1116829644879053 | 0.20482809070958302 | 0.15985576923076922 |
| 0.89999999999999991 | 0.09264024704065878 | 0.13094367227505485 | 0.09735576923076923 |
| 0.99999999999999989 | 0.06999485331960885 | 0.08778346744696415 | 0.0625 |
| 1.0999999999999999 | 0.06176016469377252 | 0.07461594732991954 | 0.043870192307692304 |
| 1.2 | 0.04014410705095214 | 0.04242867593269934 | 0.028245192307692308 |
| 1.3 | 0.024189397838394237 | 0.026335040234089245 | 0.018629807692307692 |
| 1.4000000000000001 | 0.01544004117344313 | 0.013899049012435992 | 0.01141826923076923 |
| 1.5000000000000002 | 0.0051466803911477095 | 0.008046817849305048 | 0.007211538461538462 |
| 1.6000000000000003 | 0.0066906845084920225 | 0.005120702267739576 | 0.0078125 |
| 1.7000000000000004 | 0.002058672156459084 | 0.002926115581565472 | 0.0018028846153846155 |
| 1.8000000000000005 | 0.001544004117344313 | 0.0021945866861741038 | 0.0018028846153846155 |
| 1.9000000000000006 | 0.001544004117344313 | 0.000731528895391368 | 0.0006009615384615385 |
| 2.0000000000000004 | 0.001029336078229542 | 0.001463057790782736 | 0.0006009615384615385 |Normalised Frequency
Distance (µm)
B-1
B-2
B-3
HEC-1B
B-1
B-2
B-3
A-1
A-2
A-3
HEC-1A
A-1
A-3
A-2
Supplementary Fig. S3 L-selectin binding to HEC-1B and HEC-1A endometrial cell surface models (B1-3 & A1-3) through AFM SMFS, resulting in measurements of distance (µm). SMFS data shown as box plots and frequency histograms for a minimum of 8000 curves, 5 cells per sample and minimum of 3 biological repeats

## Slide 5
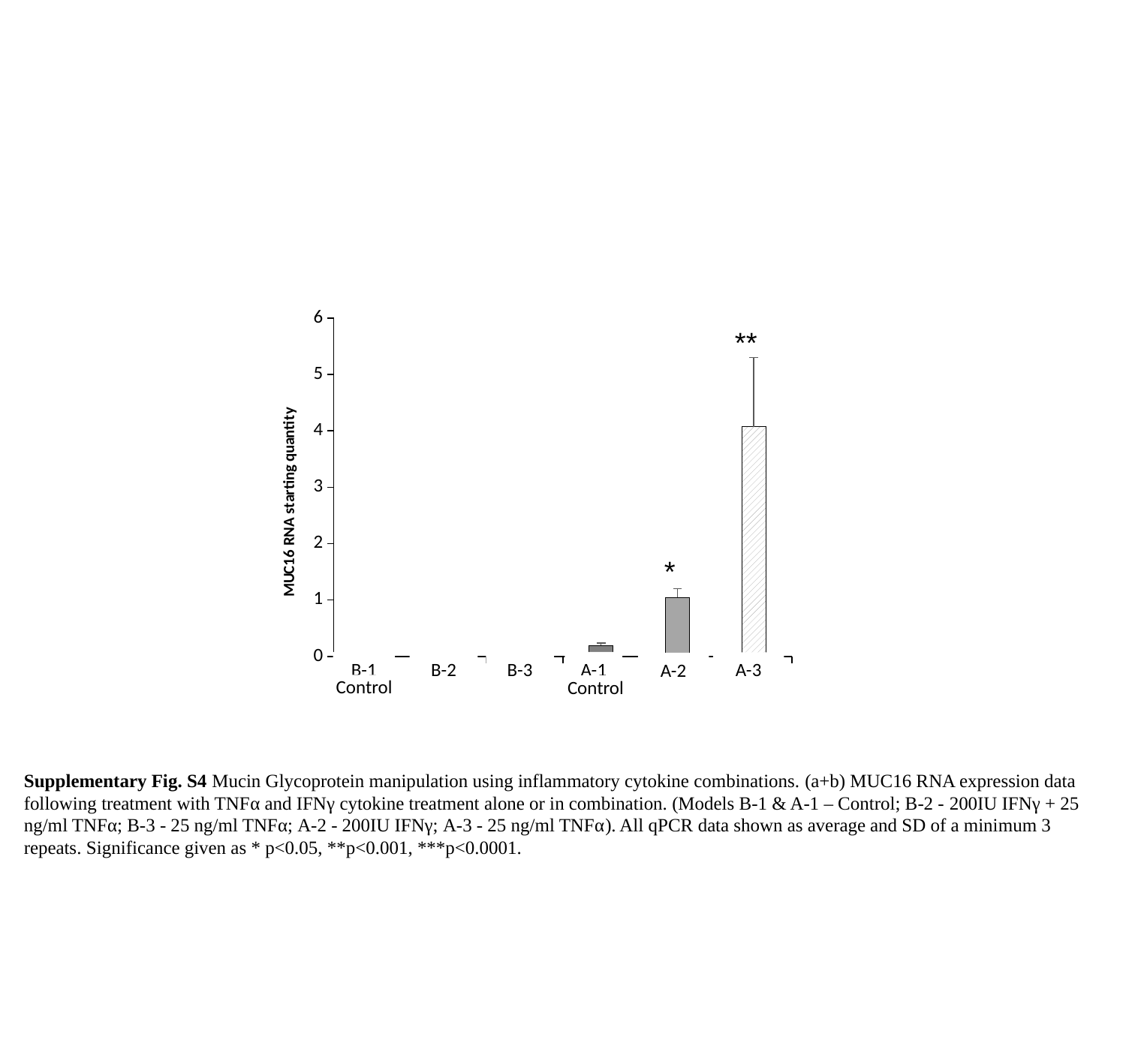

### Chart
| Category | |
|---|---|
| HEC 1B Control | 0.00536 |
| Model 1 | 0.01523 |
| Model 2 | 0.022 |
| HEC 1A Control | 0.194414 |
| Model 3 | 1.0424 |
| Model 4 | 4.0723 |**
*
A-1
B-1
B-2
B-3
A-3
A-2
Control
Control
Supplementary Fig. S4 Mucin Glycoprotein manipulation using inflammatory cytokine combinations. (a+b) MUC16 RNA expression data following treatment with TNFα and IFNγ cytokine treatment alone or in combination. (Models B-1 & A-1 – Control; B-2 - 200IU IFNγ + 25 ng/ml TNFα; B-3 - 25 ng/ml TNFα; A-2 - 200IU IFNγ; A-3 - 25 ng/ml TNFα). All qPCR data shown as average and SD of a minimum 3 repeats. Significance given as * p<0.05, **p<0.001, ***p<0.0001.

## Slide 6
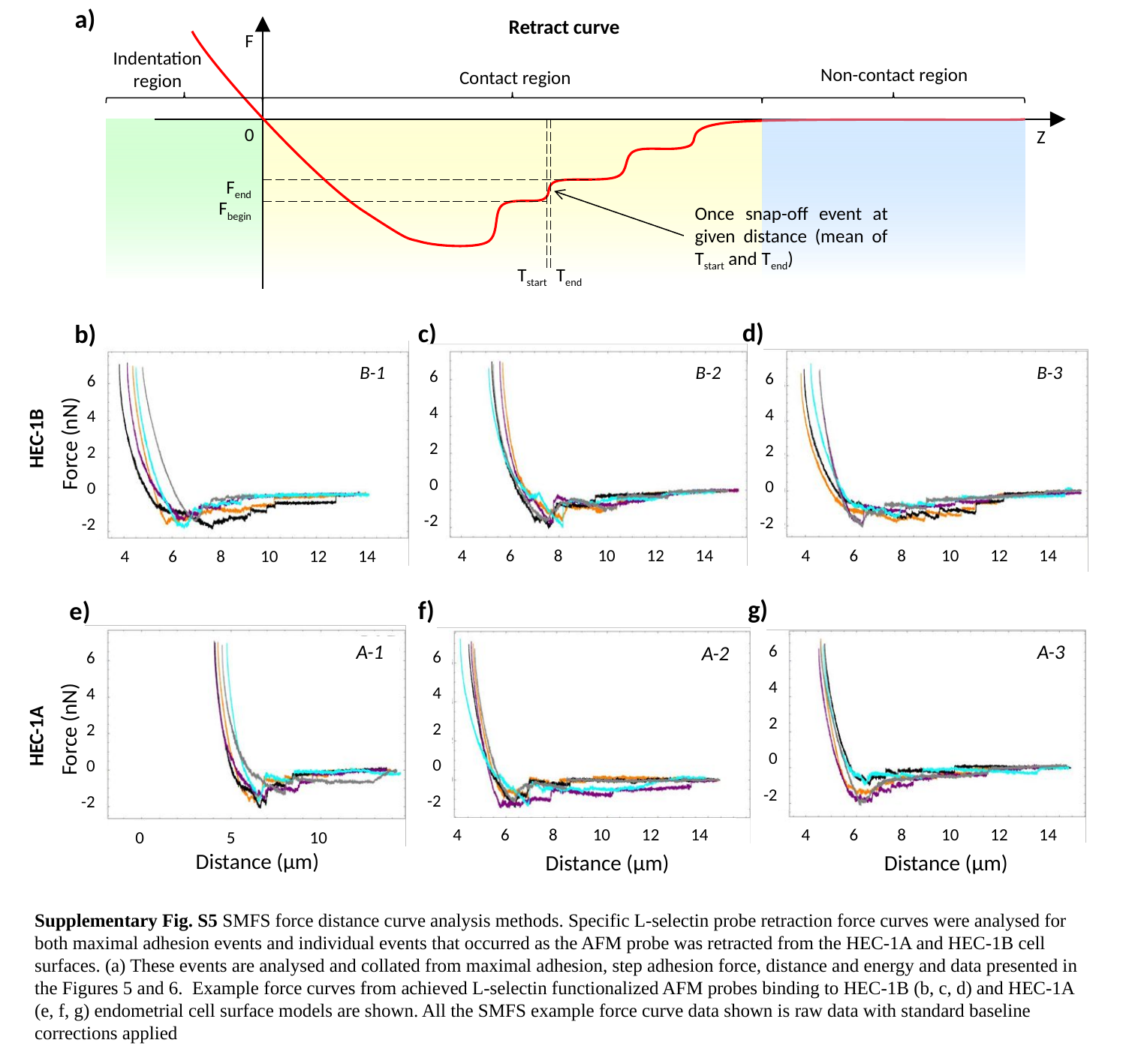

a)
Retract curve
F
Indentation region
Non-contact region
Contact region
0
Z
Fend
Fbegin
Once snap-off event at given distance (mean of Tstart and Tend)
Tstart
Tend
d)
c)
b)
HEC-1B
g)
f)
e)
A-3
A-1
A-2
HEC-1A
B-2
B-3
B-1
6
4
2
0
-2
6
4
2
0
-2
6
4
2
0
-2
Force (nN)
4 6 8 10 12 14
4 6 8 10 12 14
4 6 8 10 12 14
6
4
2
0
-2
6
4
2
0
-2
6
4
2
0
-2
Force (nN)
4 6 8 10 12 14
4 6 8 10 12 14
 0 5 10
Distance (µm)
Distance (µm)
Distance (µm)
Supplementary Fig. S5 SMFS force distance curve analysis methods. Specific L-selectin probe retraction force curves were analysed for both maximal adhesion events and individual events that occurred as the AFM probe was retracted from the HEC-1A and HEC-1B cell surfaces. (a) These events are analysed and collated from maximal adhesion, step adhesion force, distance and energy and data presented in the Figures 5 and 6. Example force curves from achieved L-selectin functionalized AFM probes binding to HEC-1B (b, c, d) and HEC-1A (e, f, g) endometrial cell surface models are shown. All the SMFS example force curve data shown is raw data with standard baseline corrections applied
