# Supplementary material for: Highly glycosylated MUC1 mediates high affinity L-selectin binding at the human endometrial surface
Source: J Nanobiotechnology. 2021 Feb 17;19:50. doi: 10.1186/s12951-021-00793-9 (PMC7890821; doi:10.1186/s12951-021-00793-9)
Supplement: Supplementary file 2 — Additional file 2. Real time Quantitative Polymerase Chain Reaction primer sequences. [file 12951_2021_793_MOESM2_ESM.docx]

**Supplementary Methods:**

**Highly glycosylated MUC1 mediates high affinity L-selectin binding at the human endometrial surface**

Lewis W Francis, Seydou N Yao, Lydia C Powell, Sean Griffiths, Alexander Berquand, Thomas Piasecki, William Howe, Andrea S Gazze, Mary C. Farach-Carson, Pamela Constantinou, Daniel Carson, Lavinia Margarit, Deya Gonzalez and R Steven Conlan_._

**Real time Quantitative Polymerase Chain Reaction (RT-QPCR)**

| **Gene** | **NCBI acsension** | **Primer direction** | **Oligo-nucleotide sequence** | **Annealing Temp (^o^C)** | **Amplicon length (bp)** |
| --- | --- | --- | --- | --- | --- |
| RPL-19 | NM_000981.3 | F | 5’-CCTGTACGGTCCATTC-3’ | 54.3 | 144 |
|  |  | R | 5’-AATCCTCATTCTCCTCATCC-3’ |  |  |
| Mucin-1 | NM_002456.5 | F | 5’-TGGTGCTGGTCTGTGTTCTG-3’ | 54.2 | 233 |
|  |  | R | 5’-CTCGCTCATAGGATGGTAGGTA-3’ |  |  |
| Mucin-16 | AF361486 | F | 5’-TTCGCCTGCCAGTCCTAAAG-3’ | 55.0 | 283 |
|  |  | R | 5’-GTGCTGGCCATTTGCATTGA-3’ |  |  |
| GLcNAcST | AB014680 | F | 5’-ATCCGTGAGAGCCTACAGGT-3’ | 59.5 | 382 |
|  |  | R | 5’-CATTGCGTGCAGATACCACG-3’ |  |  |

Table: Primer sequences. The nucleotide sequence of each gene was acquired using a NCBI nucleotide search. Ascension numbers are shown. Primers were designed using the “Pick Primers” function and annealing temperatures and amplicon lengths are shown.
